# The *fciTABC* and *feoABI* systems contribute to ferric citrate acquisition in *Stenotrophomonas maltophilia*

**DOI:** 10.1186/s12929-022-00809-y

**Published:** 2022-04-27

**Authors:** Chun-Hsing Liao, Hsu-Feng Lu, Hsin-Hui Huang, Yu Chen, Li-Hua Li, Yi-Tsung Lin, Tsuey-Ching Yang

**Affiliations:** 1 Division of Infectious Disease, Far Eastern Memorial Hospital, New Taipei City, Taiwan; 2 Department of Medicine, National Yang Ming Chiao Tung University, Taipei, Taiwan; 3 Department of Medical Laboratory Science and Biotechnology, Asia University, Taichung, Taiwan; 4 Department of Biotechnology and Laboratory Science in Medicine, National Yang Ming Chiao Tung University, Taipei, Taiwan; 5 Department of Pathology and Laboratory Medicine, Taipei Veterans General Hospital, Taipei, Taiwan; 6 Ph.D. Program of Medical Biotechnology, Taipei Medical University, Taipei, Taiwan; 7 Division of Infectious Diseases, Department of Medicine, Taipei Veterans General Hospital, Taipei, Taiwan

**Keywords:** *Stenotrophomonas maltophilia*, Ferric citrate, Feo system, Iron homeostasis

## Abstract

**Background:**

*Stenotrophomonas maltophilia*, a member of γ-proteobacteria, is a ubiquitous environmental bacterium that is recognized as an opportunistic nosocomial pathogen. FecABCD system contributes to ferric citrate acquisition in *Escherichia coli*. FeoABC system, consisting of an inner membrane transporter (FeoB) and two cytoplasmic proteins (FeoA and FeoC), is a well-known ferrous iron transporter system in γ-proteobacteria. As revealed by the sequenced genome, *S. maltophilia* appears to be equipped with several iron acquisition systems; however, the understanding of these systems is limited. In this study, we aimed to elucidate the ferric citrate acquisition system of *S. maltophilia*.

**Methods:**

Candidate genes searching and function validation are the strategy for elucidating the genes involved in ferric citrate acquisition. The candidate genes responsible for ferric citrate acquisition were firstly selected using FecABCD of *E. coli* as a reference, and then revealed by transcriptome analysis of *S. maltophilia* KJ with and without 2,2′-dipyridyl (DIP) treatment. Function validation was carried out by deletion mutant construction and ferric citrate utilization assay. The bacterial adenylate cyclase two-hybrid system was used to verify intra-membrane protein–protein interaction.

**Results:**

Smlt2858 and Smlt2356, the homologues of FecA and FecC/D of *E. coli*, were first considered; however, deletion mutant construction and functional validation ruled out their involvement in ferric citrate acquisition. *FciA* (Smlt1148), revealed by its upregulation in DIP-treated KJ cells, was the outer membrane receptor for ferric citrate uptake. The *fciA* gene is a member of the *fciTABC* operon, in which *fciT, fciA,* and *fciC* participated in ferric citrate acquisition. Uniquely, the Feo system of *S. maltophilia* is composed of a cytoplasmic protein FeoA, an inner membrane transporter FeoB, and a predicted inner membrane protein FeoI. The intra-membrane protein–protein interaction between FeoB and FeoI may extend the substrate profile of FeoB to ferric citrate. FeoABI system functioned as an inner membrane transporter of ferric citrate.

**Conclusions:**

The FciTABC and FeoABI systems contribute to ferric citrate acquisition in *S. maltophilia*.

**Supplementary Information:**

The online version contains supplementary material available at 10.1186/s12929-022-00809-y.

## Background

Iron is an essential metal in almost all living organisms, and functions as a cofactor for proteins involved in redox chemistry and electron transport [1]. Therefore, iron deprivation is a critical host defense strategy against pathogenic bacterial invasion. Bacteria have evolved numerous mechanisms to counteract this iron limitation imposed by host cells. Two forms of iron are available in nature: ferric and ferrous iron. In response to iron-limiting conditions, bacteria synthesize and secrete iron-chelating molecules to pirate ferric ions from host cells. Iron chelators include siderophores, hemophores, and citrate [2]. These iron chelators capture ferric iron or hemin from the external environment, and the chelator-iron complex must be efficiently transported across the outer and inner membranes for iron utilization [3]. Distinct from ferric iron acquisition, ferrous iron can pass by diffusion, through porins, into the periplasm and then are transported into the cytosol via inner membrane ferrous iron transporters.

Bacteria acquire citrate-mediated iron sources in two ways. Most bacteria can directly utilize exogenously supplied ferric citrate as an iron source, to fulfill their nutritional requirements under iron-depleted conditions [4]. In addition, some bacteria synthesize and secrete citrate as a siderophore to obtain ferric iron when they encounter iron-limited stress [5–7]. Bacteria generally utilize TonB-dependent outer membrane proteins (OMPs) for ferric citrate uptake across the impermeable outer membrane. FecA is a well-known cognate OMP involved in ferric citrate uptake in several bacteria [8, 9]. However, unlike the FecA-like receptors, the inner membrane transporters for ferric citrate are poorly understood, except for *fecC/D/E* in *Escherichia coli* [10]. In the *E. coli* Fec system, ferric citrate is taken up by the outer membrane receptor (FecA) and is transported into the cytoplasm via the periplasmic protein FecB and inner membrane transport proteins FecC/D/E [11]. The post-outer membrane transport systems for ferric citrate acquisition are different in *E. coli* and *Pseudomonas aeruginosa,* despite the high similarity in identity of their outer membrane receptors (FecAs). No FecB/C/D/E homologues have been found in the ferric citrate acquisition system in *P. aeruginosa*. In contrast, citrate-mediated iron uptake is compromised in *feoB* mutants of *P. aeruginosa* [9]*,* suggesting that FeoB is involved in the post-outer membrane transport of ferric citrate. However, the underlying mechanism remains unclear.

Ferric iron is the major iron source for bacteria under aerobic conditions. However, under highly acidic and anaerobic conditions, ferrous iron transport systems are important for iron acquisition. Several bacterial ferrous iron transport systems have been described [12–14]; however, the Feo system is widely conserved among different microorganisms [15–20]. The critical member of the Feo transporter system is the inner membrane protein FeoB, which is responsible for ferrous iron transport. The simplest *feo* system, represented in *Helicobacter pylori*, is only composed of the *feoB* gene [16]. In some bacteria, the Feo systems contain additional cytoplasmic proteins, in addition to FeoB. For example, the *feo* systems of *Leptospira biflexa* and *Campylobacter jejuni* consist of *feoA* and *feoB* [19, 21]. In γ-proteobacteria, the Feo system is typically composed of three proteins, FeoA, FeoB, and FeoC; and the genes encoding these proteins generally form an operon. FeoA and FeoC are cytosolic proteins that interact with FeoB to form a complex [22, 23].

Given the lethal damage caused by excess cytoplasmic iron, iron-uptake systems must be controlled. Iron deplete conditions are generally considered as stimuli for the inducible expression of iron source acquisition systems [2]. Two regulatory mechanisms are well conserved in most Gram-negative bacteria: the transcriptional ferric uptake regulator (Fur) [24] and the surface signaling cascade [25]. The Fur protein can interact with the corepressor Fe^2+^, and repress the transcription of almost all genes related to iron uptake. When the intracellular Fe^2+^ is too low to interact with Fur, the genes repressed by the Fur-Fe^2+^ complex are derepressed [24]. A classic surface signaling cascade regulation system consists of a TonB-dependent receptor for iron uptake, an extracytoplasmic function (ECF) sigma factor, and a transmembrane protein that functions as the cognate anti-sigma factor [26]. In general, the three genes encoding receptors, sigma factors, and anti-sigma factors are organized into an operon. The surface signaling cascade involved in the regulation of ferric citrate acquisition has been reported in the *fecIRABCDE* cluster of *E. coli* and the *fecIRA* operon of *P. aeruginosa* [25, 27, 28].

*Stenotrophomonas maltophilia* is ubiquitous in the environment, particularly in soil and plant rhizospheres [29]. Furthermore, this bacterium has been recognized as an important multidrug-resistant opportunistic nosocomial pathogen [30]. To inhabit diverse environmental niches, *S. maltophilia* should have evolved several iron acquisition systems for survival. However, the systems used by *S. maltophilia* to acquire iron have been poorly reported, except the FepA system for ferri-siderophore uptake [31] and the PacIRA system for xenosiderophore uptake [32]. *S. maltophilia* is known to synthesize stenobactin, a catecholate siderophore, depending on the *entCEBBFA* gene cluster [33]. FepA is a TonB-dependent OMP receptor specific for the uptake of ferri-stenobactin [31]. We surveyed the *S. maltophilia* K279a genome [34] and found many candidate genes whose annotation are associated with iron homeostasis. Then, the proteins encoded by these candidate genes were further analyzed by blastP tool of NCBI website to find their homologues in other bacteria. We disclosed the presence of an array of genes, whose products share protein identities with the components of the known iron acquisition systems. For example, Smlt0795, Smlt2858, Smlt2210, and Smlt2211 were shown to be the homologues of *hemA*, *fecA*, *feoA*, and *feoB*, respectively. However, little is known about the acquisition of hemin, ferric citrate, and ferrous iron in *S. maltophilia*. In this study, we aimed to elucidate the citrate-mediated iron acquisition system in *S. maltophilia.* We identified that the FciTABC and FeoABI, two previously unidentified systems, are responsible for ferric citrate utilization in *S. maltophilia* under iron-depleted conditions. This is distinct from the FecIRABCDE system of *E. coli* and the FecIRA/FeoB system of *P. aeruginosa*.

## Methods

### Bacterial strains, plasmids, and primers

The primers used in this study are listed in Additional file 9: Table S1. Additional file 10: Table S2 lists the bacterial strains and plasmids used in this study.

### Construction of in-frame deletion mutants

In-frame deletion strains were constructed using double cross-over homologous recombination as described previously [35]. In brief, two DNA fragments flanking the genes of interest were amplified by PCR with the primers as indicated and then subsequently cloned into pEX18Tc to generate the mutagenic plasmids. The primers used and the resultant plasmids for mutant construction were summarized in Additional file 9: Table S1 and Additional file 10: Table S2. The pEX18Tc-derived mutagenic plasmids were transported into relevant *S. maltophilia* strains via conjugation. Integration of the deletion constructs into the chromosome was selected by resistance to norfloxacin (2.5 μg/ml) and tetracycline (30 μg/ml). The double cross-over tansconjugants were selected by 10% sucrose [35]. The correctness of mutants was verified by PCR and sequencing the mutated region.

### Construction of complementation plasmids

Genes of interest were amplified by PCR with the primers as indicated and then cloned into pRK415 under the control of *lacZ* promoter. The primers used and the resultant plasmids for complementation were summarized in Additional file 9: Table S1 and Additional file 10: Table S2. After verification by sequencing, the resultant plasmids were transported into the relevant strains via conjugation.

### Viability assay

The logarithmic phase bacterial strains tested were adjusted to a concentration of 2 × 10^5^ CFU/μl followed by tenfold serial diluted. A 5 μl volume of each dilution was spotted onto the plates as indicated. After a 24-h incubation, the growth of bacterial cells was observed.

### Reverse transcription-PCR (RT-PCR) and operon verification

DNA-free RNA was isolated from mid-log phase KJ∆Fur cells as described previously [36]. Reverse transcription was carried out using the primers FciC-C and FeoI-C (Additional file 9: Table S1), respectively. FciC-C-derived c-DNA was used as the template for PCR using the primer sets FciTQ99-F/R, FciAQ102-F/R, and FciBQ104-F/R (Additional file 9: Table S1). FeoI-C-derived c-DNA was used as the template for PCR using the primer sets FeoAQ110-F/R and FeoBQ108-F/R (Additional file 9: Table S1). The PCR products were separated by electrophoresis on a 2% agarose gel and visualized by staining with ethidium bromide.

### Bacterial adenylate cyclase two-hybrid (BACTH) assay

The *feoB* and *feoI* genes were amplified by PCR using the primer sets of 25FeoB-F/R and 18FeoI-F/R (Additional file 9: Table S1), and then subsequently cloned into vectors pKT25 and pUT18, generating recombinant plasmids pKT25-FeoB and pUT18-FeoI respectively (Additional file 10: Table S2). The *feoA* gene was amplified by PCR using the primer sets of 25FeoA18-F/R (Additional file 9: Table S1) and then subsequently cloned into vectors pKT25 and pUT18, generating recombinant plasmids pKT25-FeoA and pUT18-FeoA (Additional file 10: Table S2), respectively. The nt 649–1863 and nt 7–636 of *feoB* were amplified by PCR using the primer sets of 25FeoBt-F/25FeoB-R and 25FeoB-F/25FeoBc-R (Additional file 9: Table S1), and then subsequently cloned into vector pKT25, generating recombinant plasmids pKT25-FeoBt and pKT25-FeoBc (Additional file 10: Table S2), respectively. The *fciT* gene was amplified by PCR using the primer sets of 18FciT-F/R (Additional file 9: Table S1), and cloned into vector pUT18, generating recombinant plasmid pUT18-FciT (Additional file 10: Table S2).

The pUT18-derived and pKT25-derived plasmids as indicated were co-transformed into a *∆cya* strain (*E. coli* DHM1), and transformants were grown in LB medium supplemented with ampicillin, kanamycin, and 0.5 mM isopropyl-β-D-thiogalactopyranoside (IPTG) for 16 h at 30 °C. The overnight cultures were harvested and assayed for β-galactosidase activity. β-galactosidase assays were performed using the method of Miller [37]**.** Experiments were independently repeated at least three times.

### Real time-quantitative PCR (qRT-PCR)

RNA was prepared from logarithmic phase bacterial cells grown in LB broth without or with additives as indicated and converted to cDNA by reverse transcription. qRT-PCR was performed by the ABI Prism 7000 Sequence Detection System according to the manufacturer’s protocols. All gene expressions were normalized with the internal control of 16S rRNA since [38]. All experiments were carried out in triplicate.

## Results

### *Stenotrophomonas maltophilia* can utilize exogenously supplied ferric citrate as an iron source for growth in iron-depleted conditions

**Fig. 1:**
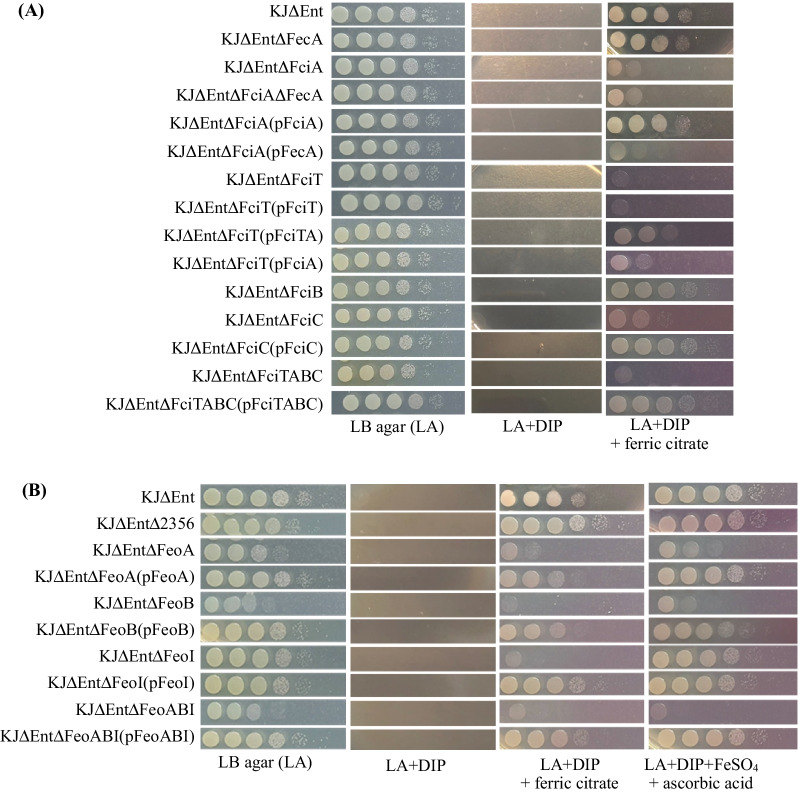
The roles of* fecA, fciTABC* operon, Smlt2356, and *feoABI* operon in ferric citrate acquisition in an iron-depleted condition. The logarithmic-phase bacterial cells of 2 × 10^5^ CFU/μl were tenfold serially diluted. Five microliters of bacterial suspension were spotted onto the LB agar plates as indicated. The concentrations of DIP, ferric citrate, FeSO_4_, and ascorbic acid used were 50 μg/ml, 110 μM, 70 μM, and 80 μM, respectively. The growth of bacterial cells was recorded after 24-h incubation at 37 °C. **A** Role of *fecA* and *fciTABC* operon in ferric citrate acquisition in an iron-depleted condition. **B** Roles of Smlt2356 and *feoABI* operon in the acquisition of ferric citrate and ferrous iron in an iron-depleted condition

*Stenotrophomonas maltophilia* can produce the catechol-type siderophore, stenobactin, under iron-depleted conditions [39]. Siderophores are the most effective iron chelators for bacteria to capture iron; thus, the contribution of other iron acquisition systems may be shielded by the functional siderophore system. In this study, *S. maltophilia* KJ∆Ent [32], a stenobactin-null mutant, was used as the parental strain to assess citrate-mediated iron acquisition. For the convenience of investigating the involvement of target genes in ferric citrate acquisition, we intended to establish an iron-depleted condition, at which the bacterial strains tested were not able to grow, and found that LB agar supplemented with 50 μg/mL 2,2′-dipyridyl (DIP) was feasible (Fig. 1). The growth of KJ∆Ent was inhibited in DIP-supplemented media and was restored in DIP- and ferric-citrate supplemented media (Fig. 1A), indicating that *S. maltophilia* can utilize ferric citrate as an iron source for growth, and its genome should contain the necessary genetic components for ferric citrate utilization.

### Smlt2858 (FecA) is not the cognate receptor for the uptake of ferric citrate

**Fig. 2:**
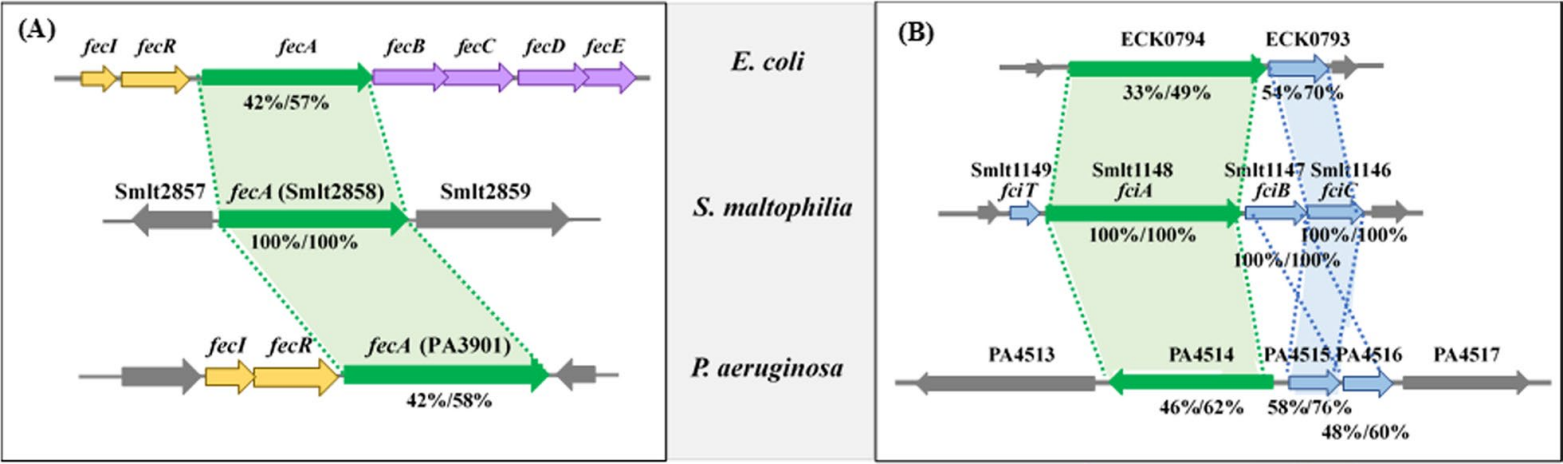
Comparison of the possible ferric citrate uptake systems among *S. maltophilia*, *E. coli*, and *P. aeruginosa*. **A** The genetic organizations of *fecIRABCED* operon of *E. coli*, *fecIRA* operon of *P. aeruginosa*, and their *fecA* homolog in *S. maltophilia*. Genes encoding for FecA are marked in green. The numbers labelled below the arrows indicate the protein identities and similarities compared to the *S. maltophilia* FecA protein. **B** The genetic organizations of *fciTABC* operon of *S. maltophilia* and its homologs in *E. coli* and *P. aeruginosa*. Genes encoding for FciA and its homologues are marked in green. The numbers labelled below the arrows indicate the protein identities and similarities compared to the *S. maltophilia* homologs

To utilize ferric citrate as an iron source, a cognate TonB-receptor is a prerequisite, such as FecA in *E. coli* and *P. aeruginosa* [9, 27, 40]. Thus, we carried out an in silico whole genome-wide survey of *S. maltophilia* K279a using *E. coli* FecA as a query. A homologue of FecA, Smlt2858, was identified. The Smlt2858 protein displayed 42% identity and 57% similarity to the FecA protein of *E. coli*, as well as 42% identity and 58% similarity to the FecA protein of *P. aeruginosa* (Fig. 2A). Thereafter, Smlt2858 was referred to as *fecA* hereafter. No *fecI* or *fecR* homologues were found near Smlt2858 (Fig. 2A).

To elucidate FecA involvement in ferric citrate acquisition, a deletion mutant (KJ∆Ent∆FecA) lacking *fecA* was constructed, and its growth was assessed under ferric citrate-supplemented and iron-depleted conditions. KJ∆Ent∆FecA displayed no visible growth in DIP-supplemented media and comparable growth with KJ∆Ent in DIP- and ferric citrate-supplemented media (Fig. 1A). Two possibilities were, therefore, proposed: (i) FecA is not the cognate receptor for the uptake of ferric citrate, or (ii) there is an additional ferric citrate receptor, which masks the FecA-mediated effects.

### Smlt1148 (FciA) is the cognate receptor for the uptake of ferric citrate

Given the failure to demonstrate the link between *fecA* and ferric citrate uptake, other TonB-dependent receptors were considered. It is presumably accepted that iron acquisition systems are upexpressed in an iron-depleted condition [2]. The comparative transcriptome analysis of KJ cells with and without DIP treatment was reported in our recent study [41]. There were twelve TonB-dependent outer membrane receptors, including FecA, identified at rates of more than tenfold upregulation in DIP-treated KJ cells (Additional file 11: Table S3). We, thus, constructed the eleven TonB-dependent receptor deletion mutants in KJ∆Ent (Additional file 10: Table S2) and sought to identify the isogenic mutants that, when compared to KJ∆Ent, showed compromised growth in ferric citrate- and DIP-supplemented media. Among the eleven mutants, only KJ∆Ent∆1148 (KJ∆Ent∆FciA) showed compromised viability (Fig. 1A). Thus, we designated Smlt1148 as FciA (ferric citrate), to avoid confusion with the annotated FecAs (Smlt2858 homologues) in several *S. maltophilia* genomes.

Since the TonB-dependent receptors of iron-uptake systems are highly redundant in bacteria, we reinvestigated the role of FecA in ferric citrate uptake in the *∆fciA* mutant. The deletion of *fecA* in KJ∆Ent∆FciA, yielding KJ∆Ent∆FciA∆FecA, did not further compromise growth in ferric citrate- and DIP-supplemented media (Fig. 1A). Furthermore, complementation of KJ∆Ent∆FciA with an intact *fciA* gene restored bacterial viability in ferric citrate-supplemented media; in contrast, the complementation assay with plasmid pFecA failed (Fig. 1A). Collectively, these results ruled out the involvement of FecA in ferric citrate acquisition.

The genomic organization surrounding *fciA* was surveyed in the *S. maltophilia* K279a genome. The *fciA* gene seemed to be a member of a four-gene operon Smlt1149-1148–1147-1146, designated as *fciT*, *fciA*, *fciB*, and *fciC* (Fig. 2B). The four genes were transcribed in the same orientation and were preceded by a potential binding site for Fur (Additional file 1: Fig. S1) [42]. The subcellular locations of the four proteins were predicted using CELLO v.2.5 (http://cello.life.nctu.edu.tw). The *fciT* gene encodes a 134 amino acid (aa) protein, whereby the N-terminus (13–35 aa) is predicted to be a transmembrane domain and the C-terminus (36–134 aa) is distributed in the periplasmic space. The 239 aa FciB protein was predicted to be a periplasmic protein, and FciC a cytoplasmic protein with features of the Fe(II)/2-oxoglutatate (2-OG)-dependent oxygenase superfamily. We were interested in understanding whether similar gene clusters existed in *E. coli* and *P. aeruginosa*. Following a genomic search, ECK0794 and ECK0795 of *E. coli* were found to be homologues of *fciA* and *fciC*; and PA4514, PA4515, and PA4516 of *P. aeruginosa* were homologues of *fciA*, *fciC*, and *fciB*, respectively (Fig. 2B).

The genomic organization of the *fci* region suggests that the four genes may form an operon, which may encode a system for ferric citrate acquisition in *S. maltophilia*. The presence of the *fciTABC* operon was verified by reverse transcriptase PCR (RT-PCR) (Additional file 2: Fig. S2).

### Role of the *fciTABC* operon in ferric citrate utilization

To investigate the involvement of the *fciTABC* operon in ferric citrate utilization, each gene of the *fciTABC* operon was mutated, individually or in combination, in KJ∆Ent to yield KJ∆Ent∆FciT, KJ∆Ent∆FciA, KJ∆Ent∆FciB, KJ∆Ent∆FciC, and KJ∆Ent∆FciTABC. The viability of each mutant in iron-limited and ferric citrate-supplemented media was assessed. As expected, mutants tested were unable to grow in DIP-supplemented media. All mutants, except for KJ∆Ent∆FciB, displayed compromised viability compared to that of KJ∆Ent (Fig. 1A).

Complementation experiments were conducted for KJ∆Ent∆FciT, KJ∆Ent∆FciA, KJ∆Ent∆FciC, and KJ∆Ent∆FciTABC by complementation with the pRK415-derived plasmid containing the respective deleted genes. All mutants, except for KJ∆Ent∆FciT, reverted viability to the parental strain levels in iron-limited and ferric citrate-supplemented media. Failure of the KJ∆Ent∆FciT complementation test led us to consider the occurrence of polar effects in KJ∆Ent∆FciT; thus, the *fciA*, *fciB*, and *fciC* transcript levels in KJ∆Ent∆FciT were determined by reverse transcription-quantitative PCR (qRT-PCR). The *fciA* transcript levels, but not those of *fciB* and *fciC*, decreased significantly in KJ∆Ent∆FciT compared to those in KJ∆Ent (Additional file 3: Fig. S3), indicating that inactivation of *fciT* has a polar effect on the expression of *fciA*. The polar effect was further verified by the restoration of ferric citrate acquisition in KJ∆FciT(pFciTA) (Fig. 1A). To further verify the role of *fciT* in ferric citrate uptake, KJ∆Ent∆FciT was complemented by introducing plasmids pFciTA and pFciA. KJ∆Ent∆FciT(pFciTA) displayed better viability than that of KJ∆Ent∆FciT(pFciA) in ferric citrate-supplemented media (Fig. 1A); therefore, supporting the involvement of FciT in ferric-citrate acquisition.

### fciA is highly conserved in *S. maltophilia*

The iron uptake systems of bacteria are diverse and are not always highly conserved within a species. For example, the PacIRA system, a xenosiderophore uptake system, is not well conserved among *S. maltophilia* isolates [32]. We were, therefore, curious about the intraspecific conservation of the *fciTABC* operon. To gain insight into the distribution of FciA in *S. maltophilia*, the *fciA* genes in the sequenced genomes and clinical isolates were assessed. We utilized the FciA protein sequence of KJ strain as a query to search 13 *S. maltophilia* genome sequences (strains K279a, Ab55555, AU12-09, D457, JV3, R551-3, EPM1, M30, WJ60, 5BA-1-2, MF89, SKK35, and RA8) available on the NCBI database. Except for strains D457 and RA8, the remaining 11 strains contained the *fciA* gene. The FciA proteins of the 12 strains (including the KJ strain) shared identities ranging from 91 to 100% based on pairwise sequence identity scores. In addition, the presence of the *fciA* gene in 14 *S. maltophilia* clinical isolates was determined by colony PCR using primer sets of FciAc-F/R (Additional file 9: Table S1). All isolates had *fciA*-positive PCR products (Additional file 4: Fig. S4).

To determine the evolutionary relationship between FciA and other known ferric citrate-associated TonB-dependent receptors, we performed a phylogenetic analysis. FecA (Smlt2858) was used for comparison (Additional file 5: Fig. S5). This analysis demonstrated that FecA of *S. maltophilia* is distantly related to the FecA receptors of *E. coli* MG1655 and *P. aeruginosa* PAO1. Nevertheless, *S. maltophilia* FciA, *P. aeruginosa* PA4514, and *E. coli* ECK0794 formed a phylogenetic clade distinct from the other assayed receptors. However, the exact functions of PA4514 and ECK0794 remain unknown.

### Smlt2356, a FecC/D homologue, is not involved in ferric citrate utilization

For efficient ferric citrate utilization, ferric citrate taken up by the FciA receptor must be transported into the cytosol; thus, a ferric citrate cytoplasmic membrane permease is required. However, no obvious gene encoding cytoplasmic membrane permease was found near the *fciTABC* operon. In the *fecABCDE* model of *E. coli* (Fig. 2A), FecC and FecD function as inner membrane permeases for ferric citrate transportation [10]. Therefore, we used FecC and FecD as queries to search for homologues in the *S. maltophilia* K279a genome, and Smlt2356 was identified as a candidate. The Smlt2356 homologue is a 347 aa inner membrane protein, which exhibited 34% identity and 52% similarity to FecC, as well as 35% identity and 54% similarity to FecD. The genomic organization surrounding Smlt2356 further supported its involvement in iron-complex utilization. The Smlt2356 gene forms part of a six-gene cluster, Smlt2353-2358, which encodes periplasmic esterase (Smlt2353), periplasmic ATP-binding protein (Smlt2354), periplasmic transport lipoprotein (Smlt2355), the FecC/D family inner membrane protein (Smlt2356), cytoplasmic protein (Smlt2357), and periplasmic protein (Smlt2358). It was, therefore, important to investigate the involvement of Smlt2356 in ferric citrate utilization; thus, we assessed the viability of KJ∆Ent∆2356 in ferric citrate- and DIP-supplemented media. KJ∆Ent∆2356 displayed viability comparable to that of KJ∆Ent (Fig. 1B), tentatively ruling out the involvement of Smlt2356 in ferric citrate utilization in *S. maltophilia*. Thus, in *S. maltophilia*, the genes associated with the transport of ferric citrate across the inner membrane are located elsewhere in the genome; in contrast to *E. coli* where, operons are composed of the genes associated with the transport of ferric citrate across the outer and inner membranes.

### *feoA*,* feoB*, and *feoI* are required for ferric citrate acquisition

FeoB, a well-known ferrous iron inner membrane transporter, has been reported to be involved in citrate-mediated iron acquisition [9, 16, 21]. Therefore, we sought to explore whether FeoB participates in ferric citrate acquisition in *S. maltophilia*.

A survey of the *S. maltophilia* K279a genome [34] revealed the presence of two genes, Smlt2210 and Smlt2211, whose products share protein identities with the FeoABC systems of different bacteria (Additional file 6: Fig. S6) and were, therefore, designated as FeoA and FeoB, respectively. However, it is worth mentioning that the protein encoded by Smlt2212 is predicted as an inner-membrane protein by CELLO v.2.5: subcellular Localization predictor (http://cello.life.nctu.edu.tw/); whereas, the FeoC proteins characterized in other bacteria, such as *E. coli*, *P. aeruginosa*, and *V. cholera*, are cytoplasmic proteins [43, 44]. To distinguish Smlt2212 from the previously known FeoC proteins, we designated Smlt2212 as FeoI (inner membrane). Our assessment showed that: (i) FeoA is an 84 aa cytoplasmic protein; (ii) FeoB is an inner membrane transmembrane protein consisting of a hydrophilic N-terminus, containing G protein-like motifs, and a hydrophobic C-terminus, composed of 11 transmembrane segments; and (iii) Predicted by TMHMM Server v. 2.0 (https://services.healthtech.dtu.dk/service.php?TMHMM-2.0), FeoI spans the inner membrane through a single transmembrane helix flanked by cytoplasmic- and periplasmic-orientated moieties at the N and C termini. The *feoABI* cluster appeared to have an operonic structure. To test this hypothesis, we performed RT-PCR and verified the presence of the *feoABI* operon (Additional file 7: Fig. S7).

To determine whether the *feoABI* operon is involved in ferric citrate utilization, in-frame deletions in *feoA*, *feoB*, or *feoI* were introduced into the chromosome of KJ∆Ent to generate the deletion mutants KJ∆Ent∆FeoA, KJ∆Ent∆FeoB, and KJ∆Ent∆FeoI. The *feoABI* operon deletion mutant KJ∆Ent∆FeoABI was also prepared. Bacterial viability was assessed to determine whether growth was affected by *feo* inactivation. Compared to that of KJ∆Ent, the viabilities of all the mutants, except KJ∆Ent∆FeoI, were slightly compromised in LB agar (Fig. 1B). Furthermore, all the mutants displayed significantly compromised viabilities compared to that of the parental strain in ferric citrate-containing and iron-depleted media. Moreover, the viabilities were almost restored to that of the parental strain when the deleted genes were complemented (Fig. 1B). This indicated that each member of the *feoABI* system contributes to ferric citrate acquisition under iron-limited conditions.

In addition to ferric citrate, the role of the *feoABI* operon in ferrous iron acquisition was also investigated, as the *feo* system is a well-known ferrous iron acquisition system in several microorganisms [45]. Ferrous iron utilization was studied using ascorbate-reduced and FeSO_4_-containing media. Inactivation of *feoA* or *feoB* from the chromosome of KJ∆Ent significantly compromised cell viability in FeSO_4_-containing media, and the viabilities were restored when the deleted gene were *in-trans* complemented (Fig. 1B). Interestingly, KJ∆Ent∆FeoI displayed viability comparable to that of KJ∆Ent (Fig. 1B), indicating that the loss of *feoI* has no negative impact on FeSO_4_ acquisition. Collectively, *feoA* and *feoB*, but not *feoI*, are required for ferrous iron acquisition.

### Intra-membrane protein–protein interactions occur between FeoB and FeoI

**Fig. 3:**
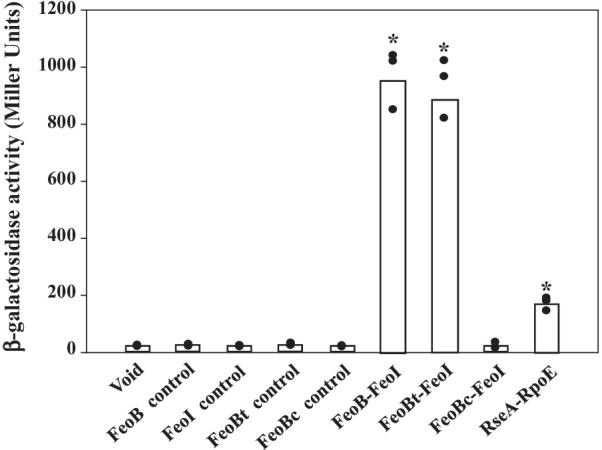
Protein–protein interaction assessed by bacterial adenylate cyclase two-hybrid (BACTH) system. Beta-galactosidase activity was determined in *E. coli* DHM1 strain coexpressing pUT18- and pKT25-derived plasmids. The pUT18- and pKT25-derived plasmids as indicated were co-transformed into *E. coli* DHM1. The transformants were grown in LB broth with ampicillin, kanamycin and IPTG for 16 h. Beta-galactosidase activity was determined and expressed as Miller units. Black dots represent the results of three independent experiments. Each bar represents the mean value of three independent experiments. *, *P* < 0.01, significance calculated by Student’s *t* test. White bars: Void, pUT18 & pKT25; FeoB control, pKT25-FeoB & pUT18; FeoI control, pKT25 & pUT18-FeoI; FeoBt control, pKT25-FeoBt & pUT18; FeoBc control, pKT25-FeoBc & pUT18. Black bars: FeoB-FeoI, pKT25-FeoB & pUT18-FeoI; FeoBt-FeoI, pKT25-FeoBt & pUT18-FeoI; FeoBc-FeoI, pKT25-FeoBc & pUT18-FeoI. Gray bar: RseA-RpoE, pKT25-RseA & pUT18-RpoE (as a positive control)

These previous results suggested that the FeoB substrate profile could be modulated by FeoI. Given that FeoI is predicted as an inner membrane protein, we speculated that there was an intermolecular interaction between FeoB and FeoI proteins. The bacterial adenylate cyclase two-hybrid system [46] was used to test this hypothesis. First, FeoB was translationally fused in frame with T25 on its N-terminus (pKT25-FeoB) and FeoI was translationally fused in frame with T18 on its C-terminus (pUT18-FeoI). The *E. coli* strain DHM1 co-expressing pKT25-FeoB and pUT18-FeoI expressed an approximately 42-fold higher β-galactosidase activity level than those in the control strains (Fig. 3). A similar procedure was used for the assessment of FeoA-FeoB and FeoA-FeoI protein–protein interactions, and no associations were detected (Additional file 8: Fig. S8). Second, to further localize the interactive region between FeoB and FeoI, the transmembrane region (aa 217–621) and cytosolic region (aa 2–212) of FeoB were individually cloned into pKT25 to yield pKT25-FeoBt (containing the transmembrane region of FeoB) and pKT25-FeoBc (containing the cytosolic region of FeoB). Significant β-galactosidase activity levels were detected in the *E. coli* strain DHM1 co-expressing pKT25-FeoBt and pUT18-FeoI, but not in the *E. coli* strain DHM1 co-expressing pKT25-FeoBc and pUT18-FeoI (Fig. 3). This result further supported that FeoI is an inner membrane protein and an intra-membrane protein–protein interaction occurs between FeoB and FeoI.

Since FciT is also predicted as an inner member protein, we wondered whether similar intra-membrane protein–protein interactions also occur between FeoB and FciT. A similar procedure was applied; however, no significant β-galactosidase activity level was detected in the *E. coli* strain DHM1 co-expressing pKT25-FeoB and pUT18-FciT (Additional file 8: Fig. S8).

### Regulation of *fciTABC* and *feoABI* expression

**Fig. 4:**
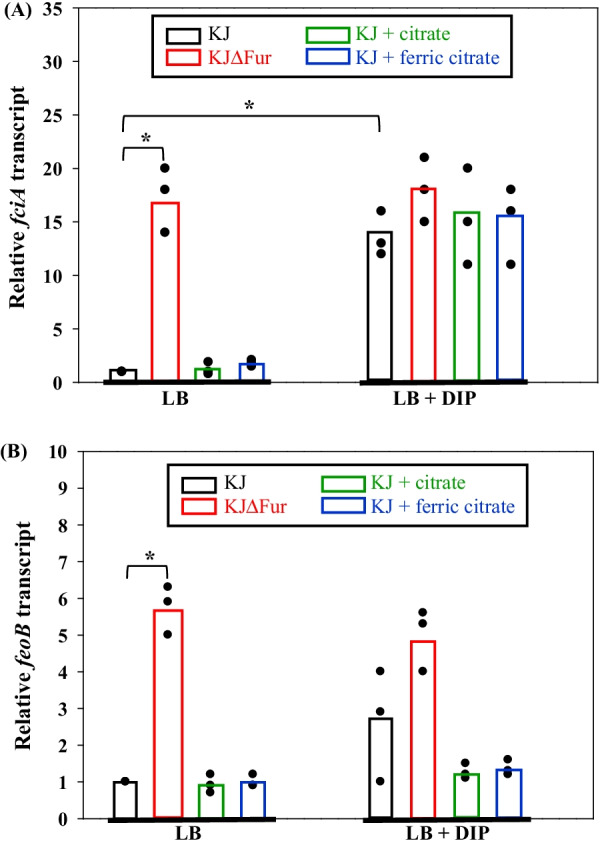
Regulation of* fciA* and *feoB* expression. Overnight culture of *S. maltophilia* strains tested was inoculated into fresh LB without or with the additives as indicated at an initial OD_450nm_ of 0.15. The *fciA* transcript (**A**) and *feoB* transcript (**B**) were quantified by qRT-PCR after a 5-h incubation. The concentrations of additives were 30 μg/ml for DIP, 1 mM for citrate, and 1 mM for ferric citrate. The relative transcript level was calculated using the transcript level of KJ cells grown in LB broth as 1. Black dots represent the results of three independent experiments. Bars represent the mean from three independent experiments. *, *P* < 0.01, significance calculated by Student’s *t* test

The impact of Fur, iron depletion, citrate, and ferric citrate on *fciA* and *feoB* expression was investigated by determining the *fciA* and *feoB* transcript levels under iron replete and iron-depleted conditions. Inactivation of *fur* resulted in an approximately 17.3-fold upregulation of *fciA* transcript; however, neither citrate nor ferric citrate played a significant role in the induction of *fciTABC* operon expression in an iron replete condition (Fig. 4A). In response to DIP challenge, *fciA* transcript of KJ cells had a 13.6-fold increment and this upregulation level was not further significantly enhanced by the treatment of citrate or ferric citrate (Fig. 4A). As for the *feoB* transcript, it was moderately upregulated in the *fur* mutant (5.7-fold increment), but no significant changes were observed in response to the challenge of DIP, citrate, and ferric citrate, either alone or combined (Fig. 4B).

## Discussion

**Fig. 5:**
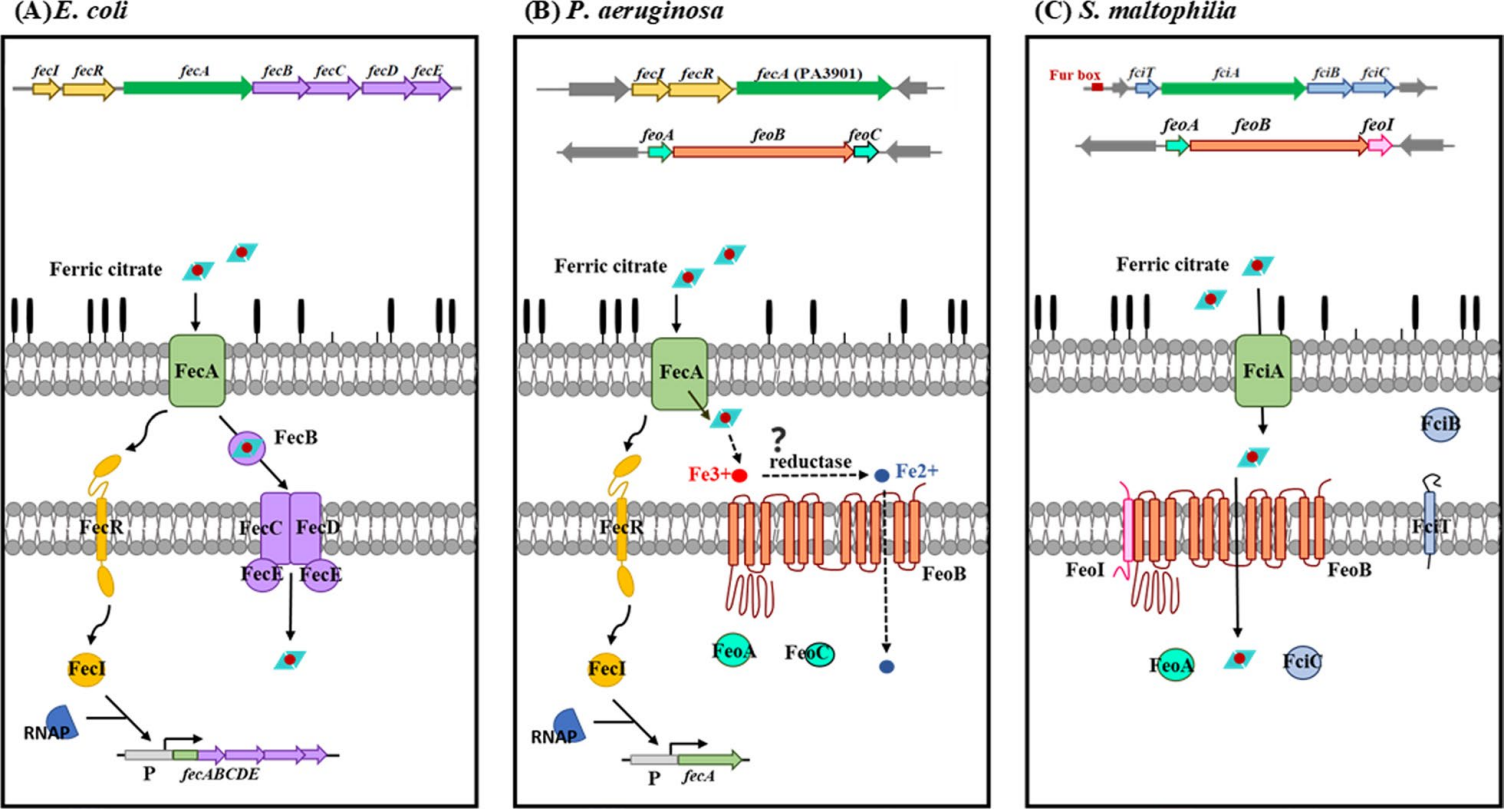
Comparisons of the ferric citrate acquisition systems between *E. coli*, *P. aeruginosa*, and *S. maltophilia*. **A** The ferric citrate acquisition system of *E. coli*. Ferric citrate is translocated into the periplasm through the TonB-dependent outer membrane receptor FecA. Meanwhile, the ferric citrate-FecA interaction results in a signal transmission from FecA, via the inner membrane-spanning FecR, to the cytoplasmic sigma factor FecI. FecI then directs the expression of the *fecABCDE* operon. In the periplasm, FecB shuttles ferric citrate into the cytoplasm through the inner membrane transport system FecC/D/E. **B** The ferric citrate acquisition system of *P. aeruginosa*. Ferric citrate is translocated into the periplasm via FecA and initiates the signaling transduction via FecR to FecI. FeoB is an inner membrane transporter responsible for citrate-mediated iron acquisition. The model suggests that the ferric iron, released from ferric citrate, in the periplasm is reduced to ferrous iron and subsequently transported into the cytosol via FeoB. **C** The ferric citrate acquisition system of *S. maltophilia*. Ferric citrate is translocated into the periplasm via FciA. Ferric citrate is then transported across the inner membrane via the FeoABI inner membrane transporter system. The *fciA* gene is a member of the *fciTABC* operon. FciT and FciC, but not FciB, participate in citrate-mediated iron acquisition. FeoI can modulates the FeoB-mediated ferric citrate transportation via an intra-membrane protein–protein interaction

Citrate-promoted iron acquisition in several bacteria has been recognized for some time, and FecA is a well-studied outer membrane receptor for ferric citrate acquisition in *E. coli* and *P. aeruginosa* [8, 9] (Fig. 5A, B). The *E. coli* FecA protein (FecA_Ec_) and *P. aeruginosa* FecA protein (FecA_Pa_) share 63% identity and 73% similarity. Subsequently, *fecA* homologous genes have been annotated in many sequenced bacterial genomes based on their encoded protein sequences that are identical to FecA_Ec_ and FecA_Pa_. However, the exact functions of these annotated FecA proteins have not yet been clearly elucidated. FecA_Sm_ (Smlt2858) was the first candidate considered as the ferric citrate receptor in *S. maltophilia*, as it demonstrated the highest identity to FecA_Ec_ and FecA_Pa_. Nevertheless, the functional investigation of FecA_Sm_ did not reveal its role in ferric citrate acquisition (Fig. 1A). However, we demonstrated that FciA (Smlt1148) is the main receptor for ferric citrate acquisition in *S. maltophilia* KJ (Fig. 1A) even though the protein sequence identity between FciA and FecA_Ec_ was not as high as that between FecA_Sm_ and FecA_Ec_. The functional studies of FciA and FecA_Sm_ exemplify the limits of assigning protein functions using bioinformatic methods. A similar result was observed in Smlt2356 and *E. coli* FecC/D (Fig. 1B). Phylogenetic analysis revealed that FecA_Sm_, rather than FciA, was phylogenetically closer to FecA_Ec_ and FecA_Pa_, and *S. maltophilia* FciA, *E. coli* ECK0794, and *P. aeruginosa* PA4514 were phylogenetically clustered (Additional file 5: Fig. S5). The functions of ECK0794 and PA4515 are unclear; thus, the significance of the FciA/ECJ0794/PA4515 phylogenetic cluster cannot be concluded. However, we can conclude that FciA in *S. maltophilia* is a novel TonB-dependent OMP receptor responsible for the uptake of ferric citrate.

The best understood post-outer membrane transport system for ferric citrate in Gram-negative bacteria is the *fecBCDE* system of *E. coli*; whereby, periplasmic ferric citrate is carried by periplasmic protein FecB and subsequently transported into the cytoplasm via the inner membrane transporter system FecC/D/E [10] (Fig. 5A). Additionally, the involvement of *FeoB* in post-outer membrane ferric citrate acquisition has been proposed in *H. pylori, L. biflexa,* and *P. aeruginosa* [9, 16, 21]. However, the *feo* systems of these microorganisms are different from that of *S. maltophilia*. The *feo* systems of *H. pylori, L. biflexa,* and *P. aeruginosa* are composed of *feoB*, *feoAB*, and *feoABC*, respectively. In particular, the FeoC of *P. aeruginosa* is a cytoplasmic protein that is distinct from the FeoI of *S. maltophilia*, which is predicted as an inner-transmembrane protein. Unfortunately, the role of FeoC in ferric citrate acquisition has not yet been investigated in *P. aeruginosa* [9]. In the known *feoABC* system of *S. enterica*, FeoC is a cytoplasmic protein that binds and protects the FeoB transporter from FtsH-mediated proteolysis [43]. Furthermore, FeoC possesses an Fe-S cluster-binding site, which may make it oxygen-sensitive and susceptible to degradation by Lon protease under high-oxygen conditions [47]. However, an Fe-S cluster-binding site was not identified in *S. maltophilia* FeoI. No evidence supports the involvement of the FeoABC system in citrate-mediated iron acquisition in *S. enterica*. Before this study, ferrous iron was thought to be the sole substrate for the FeoB transporter. Proposed models in *H. pylori* and *P. aeruginosa* suggest that the ferric iron, released from ferric citrate, is reduced to ferrous iron in the periplasm and subsequently transported into the cytoplasm via FeoB [9, 16] (Fig. 5B). In this study, we revealed a novel *feo* system, FeoABI, in *S. maltophilia*, which can transport both ferrous iron and ferric citrate. Inactivation of *feoI* significantly compromised stenobactin-null KJ cells (KJ∆Ent) to utilize ferric citrate, but not ferrous iron, as the iron sources to support growth in an iron-depleted condition (Fig. 1B), supporting that FeoI displays a critical role in modulating the substrate profile of FeoB transporter. FeoI seems to exert its function via intra-membrane protein–protein interactions with FeoB to extend the ability of FeoB to transport ferric citrate. Thus, our results support that ferric citrate is a compatible substrate for the FeoABI system in *S. maltophilia*. However, the possibility that iron is dissociated from citrate in periplasm and then transported via FeoB as ferrous iron was not immediately ruled out, even though it seems not to be the dominant way.

Given that balanced iron levels are critical for bacterial survival, it has been proposed that the citrate-mediated iron acquisition systems of *E. coli* and *P. aeruginosa* are induced in the conditions of iron limitation and ferric citrate availability [4, 9]. The iron limitation alone is not enough to induce the *fecA* expression in *P. aeruginosa* [9]. However, in this study, we found that iron limitation alone was enough to upregulate *fciA* expression and the presence of citrate or ferric citrate hardly further enhanced its expression in *S. maltophilia* (Fig. 4A). Collectively, citrate and ferric citrate seem not to play a significant role in the *fciTABC* induction of *S. maltophilia*, unlike their involvement in the *fecA* induction of *E. coli* and *P. aeruginosa* [4, 9].

The surface signaling cascade and Fur are known key regulatory mechanisms for iron utilization in several bacteria [24, 25]. Furthermore, the expression of ferric citrate acquisition components, in *E. coli* and *P. aeruginosa*, is regulated by the surface signaling cascade composed of *fecA* and *fecI*, that encode an extracytoplasmic function sigma factor; and *fecR*, that encodes the cognate anti-sigma factor [25, 27], (Fig. 2A). However, homologues of *fecI* and *fecR* were not found in the vicinity of *fciA* (Fig. 2B), suggesting that the ferric citrate transport system in *S. maltophilia* may not be regulated by the surface signaling cascade, or the regulators could be located elsewhere in the genome.

## Conclusion

In response to iron-depleted stress, *S. maltophilia* is able to utilize ferric citrate as the sole iron source for growth. Figure 5C presents a ferric citrate acquisition model that concludes the experimental results of this study. Under iron-depleted conditions, ferric citrate is taken up by FciA, a TonB-dependent outer membrane protein, and then mainly transported across the inner membrane via the FeoABI system. The *fciA* is a member of *fciTABC* operon, in which *fciT, fciA,* and *fciC* contribute to ferric citrate acquisition. The FeoABI system functions as an inner membrane transporter for both ferrous iron and ferric citrate. FeoI is required for FeoB-mediated ferric citrate transportation but is dispensable for FeoB-mediated ferrous iron transportation. The expression of *fciTABC* and *feoABI* operons is regulated by Fur and iron limitation and less related to citrate and ferric citrate. Collectively, the FciTABC and FeoABI systems are vital for ferric citrate acquisition in *S. maltophilia*.

## Supplementary Information

**Additional file 1: Fig. S1.** Diagram of *fciTABC* operon and its promoter region in *S. maltophilia*.

**Additional file 2: Fig. S2.** *FciTABC* operon verification of *S. maltophilia*.

**Additional file 3: Fig. S3.** Polar effect assessment of KJ∆Ent∆FciT.

**Additional file 4: Fig. S4.** The prevalence of *fciA* gene in *S. maltophilia* clinical isolates.

**Additional file 5: Fig. S5.** Phylogenetic relationship between FecA and FciA of *S. maltophilia* and their homologs in other bacteria.

**Additional file 6: Fig. S6.** The genetic organization of *feoABI* operon of *S. maltophilia* and its homologues in *P. aeruginosa*, *E. coli*, and *V. cholera*.

**Additional file 7: Fig. S7.** *FeoABI* operon verification of *S. maltophilia*.

**Additional file 8: Fig. S8.** Protein–protein interaction assessed by bacterial adenylate cyclase two-hybrid (BACTH) system.

**Additional file 9: Table S1.** Primers used for the construction of pEX18Tc-derived mutagenic plasmids, complementation plasmids, operon validation, and qRT-PCR

**Additional file 10: Table S2.** Bacterial strains and plasmids used in this study

**Additional file 11: Table S3.** Transcriptomic analysis of TonB-dependent outer membrane receptor genes differentially expressed in *S. maltophilia* KJ with and without the DIP treatment

## Data Availability

Data and materials related to this study are available upon request.

## Abbreviations

DIP: 2,2′-Dipyridyl
RT-PCR: Reverse transcription-PCR
qRT-PCR: Real time quantitative PCR
