# Supplementary material for: The fciTABC and feoABI systems contribute to ferric citrate acquisition in Stenotrophomonas maltophilia
Source: J Biomed Sci. 2022 Apr 27;29:26. doi: 10.1186/s12929-022-00809-y (PMC9047314; doi:10.1186/s12929-022-00809-y)
Supplement: Supplementary file 1 — Additional file 1: Fig. S1. Diagram of fciTABC operon and its promoter region in S. maltophilia. [file 12929_2022_809_MOESM1_ESM.docx]

***Smlt1150 fciT fciA fciB fciC***

***Smlt1150***

TGA*GGCGGCGCCCGGCCCGGAGAGCCCCCAGAGGGCGCCTCGCGGGAGAGCATGCACCCATGGGGCGGATCCACTGAAGTGCACTGGGGGGCCCTCCAAGGCCCTTCCAGGCCATCCAGATCGGCAACCCGATTGCGCATATGCGAATCATTATCGTTGATGTTATTCTACGTAACATCTTCGTAACGACATCGCCCCTGCGCC*ATG

***fciT***

**Fig. S1. Diagram of *fciTABC* operon and its promoter region in *S. maltophilia*.** The orientation of gene is indicated by arrow. The putative Fur box is marked in yellow, based on the reported Fur box sequence (Garcia et al., 2015). The putative promoter region of the *fciTABC* operon is underlined (promoter prediction: <http://www.phisite.org/main/index.php?nav=tools&nav_sel=hunter>).
