# Supplementary material for: The fciTABC and feoABI systems contribute to ferric citrate acquisition in Stenotrophomonas maltophilia
Source: J Biomed Sci. 2022 Apr 27;29:26. doi: 10.1186/s12929-022-00809-y (PMC9047314; doi:10.1186/s12929-022-00809-y)
Supplement: Supplementary file 2 — Additional file 2: Fig. S2. FciTABC operon verification of S. maltophilia. [file 12929_2022_809_MOESM2_ESM.docx]

**(A)**

***Smlt1150 fciT fciA fciB fciC***

FciCQ96-F/R

FciBQ104-F/R

FciAQ102-F/R

FciTQ99-F/R

FciC-C

**(B)**

1 2 3 4 5


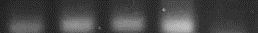


**Fig. S2. *FciTABC* operon verification of *S. maltophilia*.** (A) The genetic organization of *fciTABC* operon. The orientation of gene is indicated by arrow. The four bars below the gene indicate the locations of expected PCR amplicons. The small black arrow indicates the location of primer FciC-C. (B) Agarose gel electrophoresis of the products of RT-PCR. Overnight-cultured *S. maltophilia* KJΔFur was inoculated into fresh LB with an initial OD450 of 0.15 and grown for 5 h. The cDNAs were obtained by RT-PCR using the primer FciC-C. cDNA (100 ng) was used as the template for PCR with the primers indicated. Lane 1, primers FciTQ99-F and FciTQ99-R; Lane 2, primers FciAQ102-F and FciAQ102-R; Lane 3, primers FciBQ104-F and FciBQ104-R; Lane 4, primers FciCQ96-F and FciCQ96-R; Lane 5, primers SmeXQ-F and SmeXQ-R.
