# Supplementary material for: The fciTABC and feoABI systems contribute to ferric citrate acquisition in Stenotrophomonas maltophilia
Source: J Biomed Sci. 2022 Apr 27;29:26. doi: 10.1186/s12929-022-00809-y (PMC9047314; doi:10.1186/s12929-022-00809-y)
Supplement: Supplementary file 3 — Additional file 3: Fig. S3. Polar effect assessment of KJ∆Ent∆FciT. [file 12929_2022_809_MOESM3_ESM.docx]

**1.6**

**1.4**

**1.2**

**1.0**

**0.8**

**0.6**

**0.4**

**0.2**

**0**

KJΔEnt

KJΔEntΔFciT

*****

**Relative transcript level**

***fciA fciB fciC***

**Fig. S3. Polar effect assessment of KJΔEntΔFciT.** Overnight culture of *S. maltophilia* KJΔEnt and KJΔEntΔFciT were inoculated into fresh LB at an initial OD_450nm_ of 0.15. The *fciA, fciB,* and *fciC* transcripts were quantified by qRT-PCR after a 5-h incubation. The relative transcript level was calculated using the transcript level in KJΔEnt as 1. Black dots represent the results of three independent experiments. Bars represent the mean from three independent experiments. *, *P* < 0.01, significance calculated by Student’s *t* test.
