# Supplementary material for: The fciTABC and feoABI systems contribute to ferric citrate acquisition in Stenotrophomonas maltophilia
Source: J Biomed Sci. 2022 Apr 27;29:26. doi: 10.1186/s12929-022-00809-y (PMC9047314; doi:10.1186/s12929-022-00809-y)
Supplement: Supplementary file 4 — Additional file 4: Fig. S4. The prevalence of fciA gene in S. maltophilia clinical isolates. [file 12929_2022_809_MOESM4_ESM.docx]

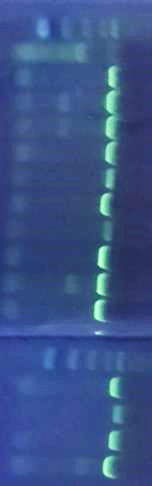


M 1 2 3 4 5 6 7 8 9 10 11 M 12 13 14 15

**500 bp**

**Fig. S4. The prevalence of *fciA* gene in *S. maltophilia* clinical isolates.** Colony PCR was performed to investigate the *fciA* prevalence in *S. maltophilia* clinical isolates. The *S. maltophilia* strains tested (KJΔFciA and 14 clinical isolates) were freshly cultured on LB agar and incubated for 24 h. The bacterial cells picked up by sterilized toothpick were used directly as the template for PCR with the primer sets of FciAc-F and FciAc-R. After cycling, 5 μl of each PCR amplicons were loaded on a 2% agarose gel stained with ethidium bromide. Lane M, molecular ladder; lane 1, KJΔFciA (as a negative control); lane 2-15, clinical isolates.
