# Supplementary material for: The fciTABC and feoABI systems contribute to ferric citrate acquisition in Stenotrophomonas maltophilia
Source: J Biomed Sci. 2022 Apr 27;29:26. doi: 10.1186/s12929-022-00809-y (PMC9047314; doi:10.1186/s12929-022-00809-y)
Supplement: Supplementary file 5 — Additional file 5: Fig. S5. Phylogenetic relationship between FecA and FciA of S. maltophilia and their homologs in other bacteria. [file 12929_2022_809_MOESM5_ESM.docx]

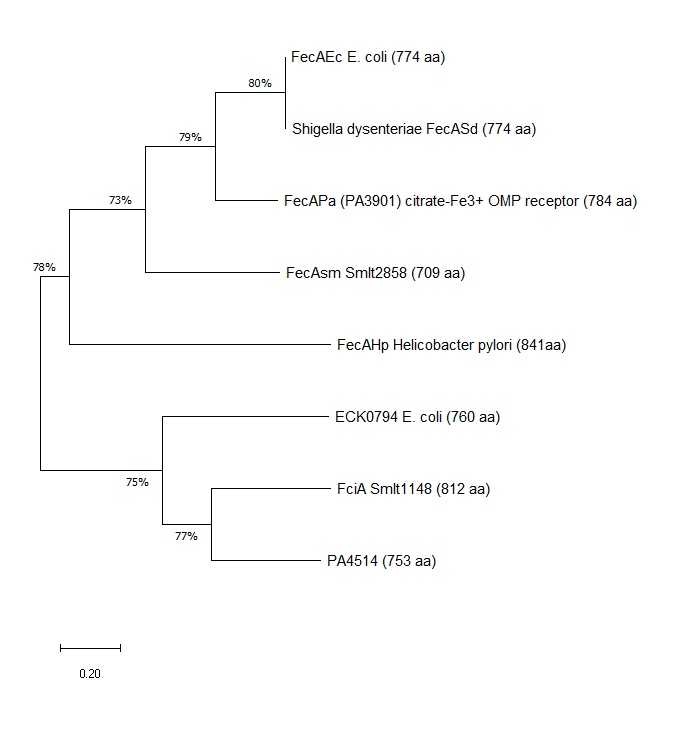


***Escherichia coli* MG1655, FecA_Ec_ (AAC77247)**

***Shigella dysenteriae* NCTC4837, FecA_Sd_ (SPZ68647)**

***Pseudomonas aeruginosa* PAO1, FecA_Pa_ (AAG07288)**

***Stenotrophomonas maltophilia* KJ, FecA_Sm_**

***Helicobacter pylori* MKM6, FecA_Hp_ (BAW78830)**

***Escherichia coli* MG1655, ECK0794 (AAC73892)**

***Stenotrophomonas maltophilia* KJ, FciA**

***Pseudomonas aeruginosa* PAO1, PA4514 (AAG07902)**

**Fig. S5. Phylogenetic relationship between FecA and FciA of *S. maltophilia* and their homologs in other bacteria.** The dendrogram was constructed with the amino acid sequences of the proteins by the neighbor-joining method. The numbers below the branches indicated the bootstrap numbers, which were calculated from 1,000 replicates.
