# Supplementary material for: The fciTABC and feoABI systems contribute to ferric citrate acquisition in Stenotrophomonas maltophilia
Source: J Biomed Sci. 2022 Apr 27;29:26. doi: 10.1186/s12929-022-00809-y (PMC9047314; doi:10.1186/s12929-022-00809-y)
Supplement: Supplementary file 6 — Additional file 6: Fig. S6. The genetic organization of feoABI operon of S. maltophilia and its homologues in P. aeruginosa, E. coli, and V. cholera. [file 12929_2022_809_MOESM6_ESM.docx]

***Smlt2210 Smlt2211 Smlt2212***

***feoA*** ***feoB*** ***feoI***

***S. maltophilia***

***P. aeruginosa***

***E. coli***

***V. cholerae***

**100%/100%** **100%/100%**  **100%/100%**

***feoC***

**23%/39%** **31%/43%**  **26%/34%**

***feoC***

**19%/40%** **29%/43%**  **17%/34%**

***feoC***

**27%/50%** **26%/42%**  **15%/29%**

**Fig. S6. The genetic organization of *feoABI* operon of *S. maltophilia* and its homologues in *P. aeruginosa*, *E. coli*, and *V. cholera*.** Genes encoding for FeoA, FeoB, and FeoI (or FeoC) are marked in green, blue, and orange, respectively. The numbers labelled below the gene indicate the protein identities and similarities compared to the ortholog of *S. maltophilia*.
