# Supplementary material for: The fciTABC and feoABI systems contribute to ferric citrate acquisition in Stenotrophomonas maltophilia
Source: J Biomed Sci. 2022 Apr 27;29:26. doi: 10.1186/s12929-022-00809-y (PMC9047314; doi:10.1186/s12929-022-00809-y)
Supplement: Supplementary file 7 — Additional file 7: Fig. S7. FeoABI operon verification of S. maltophilia. [file 12929_2022_809_MOESM7_ESM.docx]

**(A)**

***feoA*** ***feoB*** ***feoI***

FeoAQ110-F/R

FeoBQ108-F/R

FeoI-C

**(B)**

1 2 3


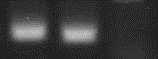


**Fig. S7. *FeoABI* operon verification of *S. maltophilia*.** (A) The genetic organization of *feoABI* operon. The orientation of gene is indicated by arrow. The two black bars below the gene indicate the locations of expected PCR amplicons. The small black arrow indicates the location of primer FeoI-C. (B) Agarose gel electrophoresis of the products of RT-PCR. Overnight-cultured *S. maltophilia* KJΔFur was inoculated into fresh LB with an initial OD_450_ of 0.15 and grown for 5 h. The cDNAs were obtained by RT-PCR using the primer FeoI-C. cDNA (100 ng) was used as the template for PCR with the primers indicated. Lane 1, primers FeoAQ110-F and FeoAQ110-R; Lane 2, primers FeoBQ108-F and FeoBQ108-R; Lane 3, primers SmeXQ-F and SmeXQ-R.
