# Supplementary material for: The fciTABC and feoABI systems contribute to ferric citrate acquisition in Stenotrophomonas maltophilia
Source: J Biomed Sci. 2022 Apr 27;29:26. doi: 10.1186/s12929-022-00809-y (PMC9047314; doi:10.1186/s12929-022-00809-y)
Supplement: Supplementary file 8 — Additional file 8: Fig. S8. Protein–protein interaction assessed by bacterial adenylate cyclase two-hybrid (BACTH) system. [file 12929_2022_809_MOESM8_ESM.docx]

**250**

**200**

**150**

**100**

**50**

**0**

*

**β-galactosidase activity (Miller Units)**

**Void**

**FeoB control**

**FeoI control**

**FeoA control**

**FciT control**

**FeoA-FeoI**

**FeoB-FeoA**

**FeoB-FciT**

**RseA-RpoE**

**Fig. S8. Protein-protein interaction assessed by bacterial adenylate cyclase two-hybrid (BACTH) system.** β-galactosidase activity was determined in *E. coli* DHM1 strain coexpressing pUT18- and pKT25-derived plasmids. The pUT18- and pKT25-derived plasmids as indicated were co-transformed into *E. coli* DHM1. The transformants were grown in LB broth with ampicillin, kanamycin and IPTG for 16 h. Beta-galactosidase activity was determined and expressed as Miller units. Black dots represent the results of three independent experiments. Each bar represents the mean value of three independent experiments. *, *P* < 0.01, significance calculated by Student’s *t* test. Void, pUT18 & pKT25; FoeB control, pKT25-FeoB & pUT18; FeoI control, pKT25 & pUT18-FeoI; FoeA control, pKT25-FeoA & pUT18. Black bars: FeoA-FeoI, pKT25-FeoA & pUT18-FeoI; FeoB-FeoA, pKT25-FeoB & pUT18-FeoA; FeoB-FciT, pKT25-FeoB & pUT18-FciT. Gray bar: RseA-RpoE, pKT25-RseA & pUT18-RpoE (as a positive control).
