# Supplementary material for: The fciTABC and feoABI systems contribute to ferric citrate acquisition in Stenotrophomonas maltophilia
Source: J Biomed Sci. 2022 Apr 27;29:26. doi: 10.1186/s12929-022-00809-y (PMC9047314; doi:10.1186/s12929-022-00809-y)
Supplement: Supplementary file 9 — Additional file 9: Table S1. Primers used for the construction of pEX18Tc-derived mutagenic plasmids, complementation plasmids, operon validation, and qRT-PCR [file 12929_2022_809_MOESM9_ESM.docx]

**Table S1 Primers used for the construction of pEX18Tc-derived mutagenic plasmids, complementation plasmids, operon validation, and qRT-PCR**

| **Primers** | **Purpose** |
| --- | --- |
| FecAN-F: CGAAGCTCCAGCGGTTGATG  FecAN-R: TGGGTACCGGTGAGGGTTGT | pΔFecA construction |
| FecAC-F: GAGGTACCTGGCCGACAAG  FecAC-R: CATCTAGAGCATTGGCTTCCA |  |
| 0795N-F: GAAGAGCTCATCGACAGCGAA  0795N-R: CGGGTACCGAAACCATCCAG | pΔ0795 construction |
| 0795C-F: GCGGTACCTCGAATGGTCGT  0795C-R: CGTCTAGAGGGTCGACAGAC |  |
| FciAN-F: CTGGAGCTCGCATGATCCCGC  FciAN-R: CAGGTACCCCGACACGATCGA | pΔFciA construction |
| FciAC-F: CAAGGTACCTGCCTCGCACTA  FciAC-R: GGTCTAGAGGGTCAGTGCCT |  |
| 1233N-F: ACGAGCTCCGTACGCTGACC  1233N-R: CCTCTAGAGGGTCTTGGCAT | pΔ1233 construction |
| 1233C-F: GGTCTAGATGCTGGTGCAGT  1233C-R: GCGGTGACATCGGCCTGCTT |  |
| FepAN-F: CTGGAGCTCCACTTCGTAAAA  FepAN-R: GTCGGTACCGGTGGCGTCGGT | pΔFepA construction |
| FepAC-F: GCGGTACCAGGTGACCGAGA  FepAC-R: CGTCTAGAGATGCGATGTAC |  |
| 1762N-F: GAGAGCTCCTGCCAGGATCT  1762N-R: GCTCTAGACGTACTGGACTG | pΔ1762 construction |
| 1762C-F: GTTCTAGATCAGCCTGCACA  1762C-R: TCGTCGACACCATCCTCGAT |  |
| PacAN-F: CAGGTACCAGGCGTCTTCGA  PacAN-R: GGTGAATTCGCCATCAACAGC | pΔPacA construction |
| PacAC-F: GGTCTAGAGGCAGTACATCAGC  PacAC-R: CCGTCGACCTTCATGTTGT |  |
| 2714N-F: GTCTCTAGACTGTTCCACCTGCAGCAAC  2714N-R: CAGGAGCTCCTCCGGAATGGTGTACT | pΔ2714 construction |
| 2714C-F: GGTGAGCTCGGTGGCTACCTGCTGAC  2714C-R: GCAGAATTCGGGAGATCATTTTCATGG |  |
| 2937N-F: GTGAGCTCTACCGAGCATCG  2937N-R: CTGGTACCAGTGGAACGGTG | pΔ2937 construction |
| 2937C-F: GGGGTACCATCGAAAACCTG  2937C-R: CATTCTAGAAGTTCCTGCAGG |  |
| 3022N-F: CCAAGCTTCTGGACAAGGT  3022N-R: GAACTGCAGCACCAGCTC | pΔ3022 construction |
| 3022C-F: TACCTGCAGGACGAACTG  3022C-R: GGTCTAGACGCGTCCACAA |  |
| 3898N-F: GCCAAGCTTGGATGATGTTCCCG  3898N-R: ACTGTCGACGCGGTCGCCGGC | pΔ3898 construction |
| 3898C-F: AACGTCGACCAGTTCAAGGTC  3898C-R: CACGAATTCGCGACCGATCAGTAC |  |
| 4135N-F: CCAAGCTTGTCCTGGCTGTA  4135N-R: CCTCTGCAGCGGCCAGGCC | pΔ4135 construction |
| 4135C-F: CTGCTGCAGTGGAAACTGAG  4135C-R: GATTCTAGACCAGGTAGGCGC |  |
| FciTN-F: GGCAAGCTTATCGTGGTATTCAACCT  FciTN-R: GCCGAGCTCCAACGTCAGCAGCAC | pΔFciT construction |
| FciTC-F CTGGAGCTCGCATGATCCCGC  FciTC-R CAGGTACCCCGACACGATCGA |  |
| FciBN-F: CACGAATTCCTATGCCTCGCACTAC  FciBN-R: CCGGTACCACACCGCGGCCAGC | pΔFciB construction |
| FciBC-F: CCTGGTACCAGCGCGCGGCCGAAG  FciBC-R: GTGAGGCCTCGGGCAGCTGC |  |
| FciCC-F: CCTGGTACCAGCGCGCGGCCGAAG  FciCC-R: GTGAGGCCTCGGGCAGCTGC | pΔFciC construction |
| FciCC-F: AGCGAGCTCAGGCCTGCTATGGGAAATGGAC  FciCC-R: GGCAAGCTTGCCTTCTCGGCTGATTT |  |
| FciTN-F: GGCAAGCTTATCGTGGTATTCAACCT  FciTN-R: GCCGAGCTCCAACGTCAGCAGCAC | pΔFciTABC construction |
| FciTC-F: AGCGAGCTCAGGCCTGCTATGGGAAATGGAC  FciTC-R: GGCAAGCTTGCCTTCTCGGCTGATTT |  |
| 2356N-F: CAGGTACCACGACTGATCCA  2356N-R: CATCTAGACCAGCAACATCGT | pΔ2356 construction |
| 2356C-F: CGTCTAGATTCGTTGGCCTGGT  2356C-R: AGAAGCTTGCCCAACAGGCT |  |
| FeoAN-F: GCGGTACCTCGCGGTTAT  FeoAN-R: AGTCTAGACAGCGTCATCT | pΔFeoA construction |
| FeoAC-F: GATCTAGATGACTGCTACT  FeoAC-R: ACAAGCTTCAGCGCCACCA |  |
| FeoBN-F: GAAAGCTTTGACTGCTACT  FeoBN-R: ACTCTAGACAGCGCCACCA | pΔFeoB construction |
| FeoBC-F: CGATCTAGAGCTGATGCTGGAACT  FeoBC-R: GCAGAATTCACCGCGATGATCAGGTACT |  |
| FeoIN-F: CGAAAGCTTGCTGATGCTGGAACT  FeoIN-R: GCATCTAGAACCGCGATGATCAGGTACT | pΔFeoI construction |
| FeoIC-F: GGTCTAGACAGCTGCGG  FeoIC-R: GGGAATTCGACTACCCGCTA |  |
| FeoAN-F: GCGGTACCTCGCGGTTAT  FeoAN-R: AGTCTAGACAGCGTCATCT | pΔFeoABI construction |
| FeoIC-F: GGTCTAGACAGCTGCGG  FeoIC-R: GGGAATTCGACTACCCGCTA |  |
| FecA-F: GTACTCGAGCTGGCCATAGG  FecA-R: ACGGTACCAGGGTCAGGTAG | pFecA construction |
| FciA-F: CCTAAGCTTGCAGTTGCATGATCC  FciA-R: CGCTCTAGAGCGCAGTTACATCAG | pFciA construction |
| FciT-F: GGCAAGCTTATCGTGGTATTCAACCT  FciT-R CAGGTACCCCGACACGATCGA | pFciT construction |
| FciTN-F: GGCAAGCTTATCGTGGTATTCAACCT  FciAC-R GGTCTAGAGGGTCAGTGCCT | pFciTA construction |
| FciB-F: CGAAGCTTTTGTTTCCGGCGATACTC  FciB-R:GTGAATTCTGAGAATGTCGGGGATGTG | pFciB construction |
| FciC-F: CGAAGCTTCGCCGCCGTCCCTCTGA  FciC-R: GGAGAATTCCCTTCTCGGCTGATTT | pFciC construction |
| FciT-F: GGCAAGCTTATCGTGGTATTCAACCT  FciC-R: GGAGAATTCCCTTCTCGGCTGATTT | pFciTABC construction |
| FeoA-F: GACCTGCAGCGGGAATGTTAGAATCGAG  FeoA-R: CTGGAATTCAGCTGGTTGAACAGTGC | pFeoA construction |
| FeoB-F GTTTCTAGATGCGGATCAGTGAAGCCA  FeoB-R CACGAATTCAGGAAAAGCAGCGTCAGT | pFeoB construction |
| FeoI-F: CGAAGCTTGGCGGCTGAT  FeoI-R: GGTCTAGAGACTACCCGCTA | pFeoI construction |
| FeoABC-F: GCTAAGCTTGATCTGCAAAGCATCGTTCA  FeoABC-R: CACGAATTCAGGAAAAGCAGCGTCAGT | pFeoABC construction |
| 25FeoA18-F: CACGTCTAGAGACGCTGTCCGA  25FeoA18-R: TAGCGGTACCGCGCGTCGTTC | pKT25-FeoA construction |
| 25FeoA18-F: CACGTCTAGAGACGCTGTCCGA  25FeoA18-R: TAGCGGTACCGCGCGTCGTTC | pUT18-FeoA construction |
| 25FeoB-F ACGGATCCGACTGCTACTGCC  25FeoB-R CGGGTACCGCGATGATCAGGT | pKT25-FeoB construction |
| 18FeoI-F: CGAAGCTTGGATGCCGGCCT  18FeoI-R: AGTCTAGATGCCGCTTCGGC | pUT18-FeoI construction |
| 25FeoBt-F GAGGATCCGGACCGCTGGCT  25FeoB-R CGGGTACCGCGATGATCAGGT | pKT25-FeoBt construction |
| 25FeoB-F: ACGGATCCGACTGCTACTGCC  25FeoBc-R: CCGGTACCTCGTCGATCTTCG | pKT25-FeoBc construction |
| 18FciT-F TGAAGCTTGGCTTCGCCCG  18FciT-R CGTCTAGACTGGCGTGCGGT | pUT18-FciT construction |
| FciAc-F: GACGCCGAAGCTGAAGAACG  FciAc-R: GTAGTCGTTGCTGGTGTTGC | Prevalence of *fciA* gene |
| FciC-C: GGGCAGCTTCACTTCGTG | RT-PCR |
| FciTQ99-F: GTGCTGCTGACGTTGCTG  FciTQ99-R: CAGCAGATCTTCAGCGTGTG | operon validation |
| FciAQ102-F: CCCCTACAGCAATCCGTTCT  FciAQ102-R: CGGTCGACCAGACCGTAGTA | operon validation,  qRT-PCR |
| FciBQ104-F: AGATGCACATGGAAGGCAAA  FciBQ104-R: CAGCATGTTCATCGCCATC | operon validation |
| FciCQ96-F: ATTTCCTGCACCCTGTTCCT  FciCQ96-R: GGGCAGCTTCACTTCGTG | operon validation |
| FeoC-C: GTACCGGGGAACTGCTTCTT | RT-PCR |
| FeoAQ110-F: GTGAAGGGCGAGGAAGTG  FeoAQ110-R: CGCTTGGCTTCACTGATCC | operon validation |
| FeoBQ108-F: CATTCTGGTGCTGGAGGAAT  FeoBQ108-R: GACAGCAGCGGGATGAAC | operon validation,  qRT-PCR |
| SmeXQ-F: TACGACCGCCGCAAGCAACC  SmeXQ-R: CAGCTCGAAGTAGTTGCGTGCC | RT-PCR |
| rDNA-F: GACCTTGCGCGATTGAATG  rDNA-R: CGGATCGTCGCCTTGGT | qRT-PCR |
