# Supplementary material for: The fciTABC and feoABI systems contribute to ferric citrate acquisition in Stenotrophomonas maltophilia
Source: J Biomed Sci. 2022 Apr 27;29:26. doi: 10.1186/s12929-022-00809-y (PMC9047314; doi:10.1186/s12929-022-00809-y)
Supplement: Supplementary file 10 — Additional file 10: Table S2. Bacterial strains and plasmids used in this study [file 12929_2022_809_MOESM10_ESM.docx]

**Table S2 Bacterial strains and plasmids used in this study**

| Strain or plasmid | Genotype or properties | Reference |
| --- | --- | --- |
| ***S. maltophilia***  KJ | A clinical *S. maltophilia* isolate | 1 |
| KJΔEnt    KJΔEntΔFecA  KJΔEntΔ0795  KJΔEntΔFciA  KJΔEntΔ1233  KJΔEntΔFepA  KJΔEntΔ1762  KJΔEntΔPacA  KJΔEntΔ2714  KJΔEntΔ2937  KJΔEntΔ3022  KJΔEntΔ3898  KJΔEntΔ4135 | *S. maltophilia* KJ mutant of *entF* and *entA* genes; *ΔentF, ΔentA*  *S. maltophilia* KJΔEnt mutant of *fecA* gene; *ΔfecA*  *S. maltophilia* KJΔEnt mutant of *Smlt0795* gene; *ΔSmlt0795*  *S. maltophilia* KJΔEnt mutant of *fciA* gene; *ΔfciA*  *S. maltophilia* KJΔEnt mutant of *Smlt1233* gene; *ΔSmlt1233*  *S. maltophilia* KJΔEnt mutant of *fepA* gene; *ΔfepA*  *S. maltophilia* KJΔEnt mutant of *Smlt1762* gene; *ΔSmlt1762*  *S. maltophilia* KJΔEnt mutant of *pacA* gene; *ΔpacA*  *S. maltophilia* KJΔEnt mutant of *Smlt2714* gene; *ΔSmlt2714*  *S. maltophilia* KJΔEnt mutant of *Smlt2937* gene; *ΔSmlt2937*  *S. maltophilia* KJΔEnt mutant of *Smlt2937* gene; *ΔSmlt3022*  *S. maltophilia* KJΔEnt mutant of *Smlt2937* gene; *ΔSmlt3898*  *S. maltophilia* KJΔEnt mutant of *Smlt2937* gene; *ΔSmlt4135* | 2  This study  This study  This study  This study  This study  This study  This study  This study  This study  This study  This study  This study |
| KJΔEntΔFecAΔFciA  KJΔEntΔFciT  KJΔEntΔFciB  KJΔEntΔFciC  KJΔEntΔFciTABC  KJΔEntΔ2356  KJΔEntΔFeoA  KJΔEntΔFeoB  KJΔEntΔFeoI  KJΔEntΔFeoABI | *S. maltophilia* KJΔEnt mutant of *fecA* and *fciA* genes; *ΔfecA*, *ΔfciA*  *S. maltophilia* KJΔEnt mutant of *fciT* gene; *ΔfciT*  *S. maltophilia* KJΔEnt mutant of *fciB* gene; *ΔfciB*  *S. maltophilia* KJΔEnt mutant of *fciC* gene; *ΔfciC*  *S. maltophilia* KJΔEnt mutant of *fciTABC* operon; *ΔfciTABC*  *S. maltophilia* KJΔEnt mutant of *Smlt2356* gene;  *ΔSmlt2356*  *S. maltophilia* KJΔEnt mutant of *feoA* gene; *ΔfeoA*  *S. maltophilia* KJΔEnt mutant of *feoB*gene; *ΔfeoB*  *S. maltophilia* KJΔEnt mutant of *feoI* gene; *ΔfeoI*  *S. maltophilia* KJΔEnt mutant of *feoABI* genes;  *ΔfeoABI* | This study  This study  This study  This study  This study  This study  This study  This study  This study  This study |
| ***E. coli*** |  |  |
| DH5a | F- φ80d/*acZΔM15* Δ(*lacZYA-argF*)*U169* *deoR recA1 endA1 hsdR17* (r_k_^-^ m_k_^+^) *phoA supE44λ* *thi-1 gyrA96 relA1* | Invitrogen |
| S17-1 | λ*pir*^+^ mating strain | 3 |
|  |  |  |
| **Plasmids** |  |  |
| pEX18Tc | *sacB oriT*, Tc^r^ | 4 |
| pRK415 | Mobilizable broad-host-range plasmid cloning vector, RK2 origin; Tc | 5 |
| pΔFecA | pEX18Tc with an internal-deletion *fecA* gene; Tc^r^ | This study |
| pΔ0795 | pEX18Tc with an internal-deletion *Smlt0795*  gene; Tc^r^ | This study |
| pΔFciA  pΔ1233  pΔFepA  pΔ1762  pΔPacA  pΔ2714  pΔ2937  pΔ3022  pΔ3898  pΔ4135  pΔ2356 | pEX18Tc with an internal-deletion *fciA* gene; Tc^r^  pEX18Tc with an internal-deletion *Smlt1233*  gene; Tc^r^  pEX18Tc with an internal-deletion *fepA* gene; Tc^r^  pEX18Tc with an internal-deletion *Smlt1762* gene; Tc^r^  pEX18Tc with an internal-deletion *pacA* gene; Tc^r^  pEX18Tc with an internal-deletion *Smlt2714* gene; Tc^r^  pEX18Tc with an internal-deletion *Smlt2937* gene; Tc^r^  pEX18Tc with an internal-deletion *Smlt3022* gene; Tc^r^  pEX18Tc with an internal-deletion *Smlt3898* gene; Tc^r^  pEX18Tc with an internal-deletion *Smlt4135* gene; Tc^r^  pEX18Tc with an internal-deletion *Smlt2356* gene; Tc^r^ | This study  This study  This study  This study  This study  This study  This study  This study  This study  This study  This study |
| pΔFeoA  pΔFeoB  pΔFeoI  pΔFeoABI | pEX18Tc with an internal-deletion *feoA* gene; Tc^r^  pEX18Tc with an internal-deletion *feoB* gene; Tc^r^  pEX18Tc with an internal-deletion *feoI* gene; Tc^r^  pEX18Tc with an internal-deletion *feoABI* genes; Tc^r^ | This study  This study  This study  This study |
| pFecA  pFciA  pFciT  pFciTA  pFciB  pFciC  pFciTABC  pFeoA  pFeoB  pFeoI  pFeoABI  pKT25-FeoB  pUT18-FeoI  pKT25-FeoA  pUT18-FeoA  pUT18-FciT  pKT25-FeoBt  pKT25-FeoBc | pRK415 with an intact *fecA* gene; Tc^r^  pRK415 with an intact *fciA* gene; Tc^r^  pRK415 with an intact *fciT* gene; Tc^r^  pRK415 with *fciT* and *fciA* genes; Tc^r^  pRK415 with an intact *fciB* gene; Tc^r^  pRK415 with an intact *fciC* gene; Tc^r^  pRK415 with an intact *fciTABC* operon; Tc^r^  pRK415 with an intact *feoA* gene; Tc^r^  pRK415 with an intact *feoB* gene; Tc^r^  pRK415 with an intact *feoI* gene; Tc^r^  pRK415 with an intact *feoABI* operon; Tc^r^  pKT25 with a translational-fusion T25-FeoB  pUT18 with a translational-fusion FeoI-T18  pKT25 with a translational-fusion T25-FeoA  pUT18 with a translational-fusion FeoA-T18  pUT18 with a translational-fusion FciT-T18  pKT25 with a translational-fusion T25-FeoB_217-621_ (the residues 217-612 of FeoB protein)  pKT25 with a translational-fusion T25-FeoB_2-212_ (the residues 2-212 of FeoB protein) | This study  This study  This study  This study  This study  This study  This study  This study  This study  This study  This study  This study  This study  This study  This study  This study  This study  This study |

1. Hu RM, Huang KJ, Wu LT, Hsiao YJ, Yang TC. 2008. Induction of L1 and L2 beta-lactamases of *Stenotrophomonas maltophilia*. Antimicrob Agents Chemother. 2008;52:1198-200.
2. Pan SY, Shih YL, Huang HH, Li LH, Lin YT, Yang TC. [The involvement of PacIRA system of *Stenotrophomonas maltophilia* in the uptake of *Pseudomonas aeruginosa* pyochelin and intraspecies competition for iron acquisition.](https://pubmed.ncbi.nlm.nih.gov/33811013/) J Microbiol Immunol Infect. 2021;23:S1684-1182(21)00052-9.
3. Simon R, O'Connell M, Labes M, Puhler A. A. Plasmid vector for the genetic analysis and manipulation of rhizobia and other Gram-negative bacteria. Methods Enzymol. 1986; 118:640-59.
4. Hoang TT, Karkhoff-Schweizer RR, Kutchma AJ, Schweizer HP. A broad-host-range Flp-FRT recombination system for site-specific excision of chromosomally-located DNA sequences: application for isolation of unmarked *Pseudomonas aeruginosa* mutants. Gene. 1998;212:77-86.
5. Keen NT, Tamaki S, Kobaysahi D, Trollinger D. Improved broad host-range plasmids for DNA cloning in gram-negative bacteria. Gene. 1998;70:191-7.
