# Supplementary material for: The fciTABC and feoABI systems contribute to ferric citrate acquisition in Stenotrophomonas maltophilia
Source: J Biomed Sci. 2022 Apr 27;29:26. doi: 10.1186/s12929-022-00809-y (PMC9047314; doi:10.1186/s12929-022-00809-y)
Supplement: Supplementary file 11 — Additional file 11: Table S3. Transcriptomic analysis of TonB-dependent outer membrane receptor genes differentially expressed in S. maltophilia KJ with and without the DIP treatment [file 12929_2022_809_MOESM11_ESM.docx]

**Table S3 Transcriptomic analysis of TonB-dependent outer membrane receptor genes differentially expressed in *S. maltophilia* KJ with and without the DIP treatment**

| Locus  Smlt | TPM^a^ | | Fold change^b^  RNAseq | Encoded protein |
| --- | --- | --- | --- | --- |
|  | **None** | **DIP** |  |  |
| 0795 | 2.51 | 1420.40 | +566.7 | TonB-dependent outer membrane receptor |
| 1148 | 7.58 | 488.70 | +64.45 | TonB-dependent outer membrane receptor |
| 1233 | 2.58 | 296.30 | +114.91 | TonB-dependent outer membrane receptor |
| 1426 | 23.29 | 2791.57 | +119.87 | FepA, ferri-stenobactin receptor |
| 1762 | 3.08 | 263.39 | +85.59 | TonB-dependent outer membrane receptor |
| 2666 | 2.51 | 114.16 | +45.43 | PacA, *P. aeruginosa* ferri-pyochelin receptor |
| 2714 | 5.38 | 3390.63 | +629.8 | TonB-dependent outer membrane receptor |
| 2858 | 3.37 | 145.31 | +43.15 | FecA |
| 2937 | 0.91 | 311.95 | +340.93 | TonB-dependent outer membrane receptor |
| 3022 | 7.46 | 629.93 | +84.42 | TonB-dependent outer membrane receptor |
| 3898 | 2.22 | 92.66 | +41.71 | TonB-dependent outer membrane receptor |
| 4135 | 16.18 | 1780.96 | +110.09 | TonB-dependent outer membrane receptor |

^a^TPM, Transcripts Per Kilobase Million

^b^Negative values represent genes that were significantly downregulated in response to DIP treatment, whereas positive values represent upregulation in response to DIP treatment.
